# Supplementary material for: High Quality Topic Extraction from Business News Explains Abnormal Financial Market Volatility
Source: PLoS One. 2013 Jun 6;8(6):e64846. doi: 10.1371/journal.pone.0064846 (PMC3675119; doi:10.1371/journal.pone.0064846)
Supplement: Data S1 — Exact pruning procedure and full description of all the topic distributions for the term “Toyota”. (PDF) [file pone.0064846.s002.pdf]

Supporting Information for Hisano et al

I. EXACT PRUNING PROCDEDURE OF STEP (2-B)

A. Topics reflecting repeated phrases

If the top 6 words in the topic distribution included more than one of these words listed below we defined the topic distribution as only reflecting repeated news phrases. Namely they are “top”, “news”, “reutersnet”, “messaging”, “double”, “click”, “words”, “moved”, “update”, “stories”, “press”, “corp”, “services”, and “trademark”.

B. Topics reflecting market words

If the top 6 words in the topic distribution included more than one of these words listed below we defined the topic distribution as only reflecting market news. Namely they are “stock”, “stocks”, “index”, “futures”, “market”, “rose”, “fell”, “buy”, “sell”, “nasdaq”, “nyse”, “dow”, “target”,all sorts of numbers, names of month (i.e. “jan”, “january”,...) and currency names (i.e. “dollar”, “yen”, “won”,...).

II. FULL DESCRIPTION OF LEARNED TOPICS FOR “TOYOTA”

We show the top 10 words for all the 100 topic distributions learned for ”Toyota”. The number in parenthis describes the probability of each word in the topic distribution. We also show the time evolution of news volume for all the 100 topics learned for ”Toyota” (Fig. S1).

Topic 0: (ethanol, 0.006) (oil, 0.006) (reuters, 0.005) (government, 0.004) (sugar, 0.004) (police, 0.004) (minister, 0.003) (president, 0.003) (thai, 0.003) (iraq, 0.003)

Topic 1: (toyota, 0.069) (recall, 0.020) (safety, 0.015) (vehicles, 0.012) (recalls, 0.008) (reuters, 0.007) (problems, 0.007) (toyoda, 0.006) (acceleration, 0.005) (million, 0.005)

Topic 2: (notified, 0.025) (sept, 0.016) (aug, 0.012) (company, 0.010) (feb, 0.010) (group, 0.009) (march, 0.008) (acquire, 0.007) (joint, 0.007) (oct, 0.007)

Topic 3: (percent, 0.038) (yen, 0.029) (nikkei, 0.013) (1, 0.010) (stocks, 0.010) (shares, 0.009) (maker, 0.009) (investors, 0.008) (rose, 0.007) (earnings, 0.007)

Topic 4: (pct, 0.106) (100, 0.053) (incr, 0.043) (kfw, 0.027) (50, 0.020) (eib, 0.020) (rabobank, 0.013) (5125, 0.010) (70, 0.008) (200, 0.008)

Topic 5: (asia, 0.010) (stocks, 0.009) (asian, 0.008) (hong-kong, 0.008) (results, 0.008) (earnings, 0.008) (top, 0.007) (data, 0.007) (singapore, 0.006) (markets, 0.006)

Topic 6: (pct, 0.110) (100, 0.064) (50, 0.030) (incr, 0.016) (9998, 0.016) (475, 0.014) (55, 0.013) (525, 0.012) (unit, 0.009) (200, 0.008)

Topic 7: (yen, 0.044) (percent, 0.033) (gmt, 0.016) (profit, 0.009) (1, 0.007) (billion, 0.007) (maker, 0.007) (company, 0.006) (corp, 0.006) (report, 0.006)

Topic 8: (percent, 0.046) (yen, 0.040) (nikkei, 0.014) (fell, 0.014) (shares, 0.011) (exporters, 0.009) (stocks, 0.009) (financial, 0.008) (market, 0.007) (lost, 0.007)

Topic 9: (china, 0.029) (car, 0.017) (year, 0.012) (market, 0.011) (auto, 0.011) (percent, 0.011) (cars, 0.010) (chinese, 0.009) (india, 0.009) (000, 0.009)

Topic 10: (pct, 0.105) (100, 0.048) (50, 0.046) (incr, 0.045) (575, 0.024) (bng, 0.020) (60, 0.017) (55, 0.013) (200, 0.013) (550, 0.012)

Topic 11: (pct, 0.095) (100, 0.037) (200, 0.026) (incr, 0.020) (tranche, 0.014) (55, 0.014) (625, 0.012) (50, 0.012) (deal, 0.011) (par, 0.011)

Topic 12: (news, 0.016) (percent, 0.016) (click, 0.011) (related, 0.011) (reuters, 0.010) (double, 0.009) (shares, 0.009) (euro, 0.008) (german, 0.008) (messaging, 0.007)

Topic 13: (pct, 0.073) (frn, 0.040) (par, 0.025) (200, 0.023) (incr, 0.020) (300, 0.016) (100, 0.015) (150, 0.014) (50, 0.012) (40, 0.009)

Topic 14: (hyundai, 0.034) (percent, 0.023) (won, 0.022) (sales, 0.015) (s-korea, 0.014) (market, 0.009) (shares, 0.009) (kia, 0.009) (seoul, 0.008) (year, 0.008)

Topic 15: (assigned, 0.074) (series, 0.037) (trust, 0.023) (f1ind, 0.022) (ptc, 0.020) (f1indso, 0.017) (loan, 0.017) (reaffirmed, 0.014) (lt, 0.012) (bbbind, 0.011)

Topic 16: (news, 0.050) (top, 0.038) (reuters, 0.022) (japan, 0.018) (stocks, 0.017) (visit, 0.014) (nikkei, 0.013) (data, 0.013) (30, 0.013) (markets, 0.012)

Topic 17: (oct, 0.044) (dec, 0.042) (nov, 0.037) (2009, 0.029) (zar, 0.023) (2010, 0.021) (sep, 0.021) (nzd, 0.018) (world-bank, 0.018) (nomura, 0.018)

Topic 18: (percent, 0.043) (yen, 0.034) (shares, 0.013) (nikkei, 0.010) (financial, 0.010) (stocks, 0.008) (rose, 0.008) (bank, 0.008) (banks, 0.008) (group, 0.007)

Topic 19: (pct, 0.108) (100, 0.062) (incr, 0.043) (eib, 0.028) (kfw, 0.020) (65, 0.017) (70, 0.015) (60, 0.014) (200, 0.014) (jul2009, 0.013)

Topic 20: (economy, 0.012) (japan, 0.012) (percent, 0.011) (yen, 0.011) (boj, 0.011) (japanese, 0.010) (japans, 0.008) (year, 0.008) (market, 0.007) (government, 0.006)

Topic 21: (na, 0.051) (hong-kong, 0.013) (china, 0.011) (firm, 0.010) (group, 0.009) (million, 0.008) (corp, 0.007) (2008, 0.007) (plans, 0.007) (company, 0.007)

Topic 22: (percent, 0.032) (yen, 0.022) (stocks, 0.009) (investors, 0.009) (market, 0.008) (nikkei, 0.008) (japans, 0.008) (shares, 0.008) (tokyo, 0.008) (maker, 0.005)

Topic 23: (07, 0.029) (pct, 0.019) (terms, 0.016) (08, 0.016) (06, 0.015) (yryr, 0.014) (2003, 0.013) (2006, 0.013) (dec, 0.012) (oct, 0.012)

Topic 24: (yen, 0.045) (percent, 0.038) (gmt, 0.016) (billion, 0.012) (profit, 0.011) (shares, 0.009) (year, 0.006) (fell, 0.006) (2, 0.005) (maker, 0.005)

Topic 25: (price, 0.031) (target, 0.030) (expected, 0.022) (issues, 0.021) (corp, 0.020) (outperform, 0.016) (etr, 0.016) (percentage, 0.015) (points, 0.015) (rating, 0.013)

Topic 26: (na, 0.029) (zar, 0.024) (2012, 0.022) (aud, 0.022) (sep, 0.020) (2011, 0.019) (daiwa, 0.019) (jul, 0.017) (jun, 0.017) (aug, 0.016)

Topic 27: (1, 0.073) (jpy, 0.037) (2, 0.024) (3, 0.016) (4, 0.014) (corporation, 0.012) (5, 0.010) (h1, 0.010) (6, 0.008) (krw, 0.006)

Topic 28: (percent, 0.016) (motor, 0.014) (year, 0.013) (sales, 0.012) (1, 0.012) (toyota, 0.011) (japan, 0.011) (motors, 0.010) (vehicles, 0.010) (heavy, 0.010)

Topic 29: (team, 0.014) (f1, 0.011) (race, 0.010) (toyota, 0.007) (prix, 0.007) (season, 0.007) (grand, 0.007) (teams, 0.006) (reuters, 0.006) (year, 0.006)

Topic 30: (pct, 0.112) (100, 0.083) (60, 0.027) (2004, 0.016) (50, 0.014) (625, 0.013) (2002, 0.012) (2003, 0.011) (75, 0.011) (250, 0.010)

Topic 31: (percent, 0.031) (shares, 0.016) (reuters, 0.014) (rm, 0.011) (reutersnet, 0.011) (messaging, 0.011) (euro, 0.006) (company, 0.005) (index, 0.005) (click, 0.005)

Topic 32: (apr, 0.028) (bonds, 0.020) (yen, 0.019) (10, 0.016) (sep, 0.016) (date, 0.016) (mizuho, 0.016) (sec, 0.016) (nomura, 0.012) (20, 0.012)

Topic 33: (percent, 0.063) (shares, 0.028) (index, 0.024) (adrs, 0.019) (rose, 0.014) (fell, 0.013) (leading, 0.012) (new-york, 0.011) (cents, 0.009) (european, 0.009)

Topic 34: (gold, 0.012) (prices, 0.011) (market, 0.009) (futures, 0.009) (percent, 0.008) (1, 0.008) (rubber, 0.007) (jgb, 0.007) (euro, 0.006) (demand, 0.005)

Topic 35: (years, 0.005) (china, 0.005) (people, 0.004) (japanese, 0.004) (workers, 0.004) (japan, 0.004) (year, 0.003) (time, 0.003) (business, 0.003) (work, 0.002)

Topic 36: (percent, 0.030) (sales, 0.019) (car, 0.018) (market, 0.016) (year, 0.012) (brand, 0.012) (european, 0.010) (cars, 0.009) (europe, 0.009) (registrations, 0.007)

Topic 37: (2010, 0.035) (gmt, 0.033) (title, 0.030) (reuters, 0.030) (timedate, 0.029) (description, 0.029) (feb, 0.022) (toyota, 0.013) (wed, 0.012) (insider, 0.010)

Topic 38: (pct, 0.062) (frn, 0.048) (100, 0.035) (incr, 0.019) (150, 0.013) (200, 0.013) (500, 0.011) (50, 0.011) (425, 0.010) (jan2006, 0.010)

Topic 39: (click, 0.018) (double, 0.014) (percent, 0.009) (report, 0.008) (market, 0.007) (oil, 0.007) (stocks, 0.006) (group, 0.006) (latest, 0.006) (euro, 0.005)

Topic 40: (pct, 0.048) (frn, 0.043) (deal, 0.017) (incr, 0.016) (500, 0.016) (par, 0.012) (100, 0.012) (tranche, 0.012) (250, 0.011) (10b, 0.010)

Topic 41: (debt, 0.015) (rating, 0.015) (million, 0.012) (moodys, 0.009) (ratings, 0.008) (fiscal, 0.008) (bonds, 0.007) (tax, 0.007) (state, 0.006) (credit, 0.006)

Topic 42: (pct, 0.094) (100, 0.041) (200, 0.028) (incr, 0.017) (625, 0.016) (55, 0.015) (tranche, 0.015) (575, 0.012) (multi, 0.011) (deal, 0.011)

Topic 43: (percent, 0.043) (yen, 0.036) (gmt, 0.011) (shares, 0.009) (rose, 0.008) (jal, 0.007) (nikkei, 0.007) (1, 0.006) (toyota, 0.006) (exporters, 0.005)

Topic 44: (pct, 0.076) (incr, 0.031) (frn, 0.021) (reoffer, 0.018) (100, 0.017) (eib, 0.013) (150, 0.013) (multi, 0.012) (tranche, 0.012) (200, 0.012)

Topic 45: (pct, 0.107) (incr, 0.019) (100, 0.017) (250, 0.012) (25, 0.011) (200, 0.011) (150, 0.011) (500, 0.009) (275, 0.008) (deal, 0.008)

Topic 46: (top, 0.021) (news, 0.015) (auf, 0.009) (weitere, 0.009) (der, 0.007) (und, 0.007) (index, 0.007) (firmen, 0.007) (unternehmen, 0.007) (die, 0.006)

Topic 47: (yen, 0.041) (percent, 0.037) (gmt, 0.015) (profit, 0.009) (shares, 0.008) (maker, 0.007) (price, 0.006) (toyota, 0.006) (forecast, 0.006) (stocks, 0.006)

Topic 48: (rec, 0.059) (000, 0.038) (sm, 0.032) (cargill, 0.019) (sb, 0.018) (mz, 0.015) (bunge, 0.015) (china, 0.014) (sbo, 0.012) (roads, 0.009)

Topic 49: (pct, 0.108) (100, 0.054) (incr, 0.043) (kfw, 0.027) (eib, 0.020) (50, 0.020) (rabobank, 0.013) (5125, 0.010) (200, 0.008) (70, 0.008)

Topic 50: (1, 0.016) (sales, 0.010) (car, 0.008) (2, 0.008) (pct, 0.007) (na, 0.007) (vehicles, 0.007) (3, 0.006) (4, 0.005) (10, 0.005)

Topic 51: (feb, 0.028) (bonds, 0.023) (sept, 0.022) (yen, 0.020) (10, 0.017) (na, 0.015) (date, 0.014) (coupon, 0.012) (mizuho, 0.012) (credit, 0.011)

Topic 52: (mln, 0.078) (10-yr, 0.021) (bln, 0.018) (bond, 0.017) (company, 0.016) (amt, 0.016) (rtgs, 0.016) (matdebt, 0.015) (mgrs, 0.015) (sales, 0.015)

Topic 53: (pct, 0.109) (incr, 0.049) (rabobank, 0.037) (50, 0.033) (100, 0.028) (75, 0.020) (45, 0.017) (cba, 0.013) (60, 0.013) (kfw, 0.012)

Topic 54: (gm, 0.029) (ford, 0.017) (chrysler, 0.014) (auto, 0.011) (billion, 0.008) (automakers, 0.008) (automaker, 0.006) (bankruptcy, 0.006) (workers, 0.006) (general-motors, 0.005)

Topic 55: (pct, 0.086) (par, 0.028) (reoffer, 0.025) (10b, 0.023) (frn, 0.022) (500, 0.018) (300, 0.010) (incr, 0.009) (200, 0.008) (475, 0.008)

Topic 56: (hybrid, 0.017) (cars, 0.016) (car, 0.013) (electric, 0.013) (vehicles, 0.011) (toyota, 0.010) (fuel, 0.008) (technology, 0.007) (battery, 0.006) (batteries, 0.005)

Topic 57: (words, 0.035) (moved, 0.022) (update, 0.019) (expect, 0.013) (1, 0.011) (2, 0.009) (business, 0.006) (600, 0.006) (gmt, 0.006) (3, 0.005)

Topic 58: (yen, 0.043) (percent, 0.035) (gmt, 0.017) (billion, 0.012) (profit, 0.011) (shares, 0.009) (year, 0.008) (forecast, 0.006) (maker, 0.006) (million, 0.006)

Topic 59: (percent, 0.034) (yen, 0.033) (nikkei, 0.021) (market, 0.011) (shares, 0.010) (rose, 0.008) (investors, 0.007) (exporters, 0.007) (average, 0.007) (3, 0.006)

Topic 60: (financial, 0.013) (crisis, 0.011) (economy, 0.010) (global, 0.009) (cut, 0.009) (banks, 0.008) (economic, 0.008) (recession, 0.007) (billion, 0.007) (percent, 0.006)

Topic 61: (japan, 0.026) (production, 0.020) (earthquake, 0.011) (plant, 0.011) (plants, 0.009) (nuclear, 0.009) (power, 0.009) (parts, 0.009) (quake, 0.008) (supply, 0.008)

Topic 62: (pct, 0.110) (incr, 0.060) (50, 0.049) (100, 0.032) (rabobank, 0.025) (60, 0.023) (bng, 0.016) (575, 0.015) (kfw, 0.015) (jan2015, 0.010)

Topic 63: (fixed, 0.019) (tap, 0.015) (westpac, 0.014) (bond, 0.014) (mln, 0.013) (rmbs, 0.012) (undisc, 0.012) (frn, 0.012) (eurobond, 0.012) (aaaa1aa, 0.011)

Topic 64: (03, 0.030) (02, 0.024) (pct, 0.018) (terms, 0.015) (2002, 0.015) (2001, 0.014) (1997, 0.013) (yryr, 0.013) (1999, 0.012) (dec, 0.012)

Topic 65: (pct, 0.107) (100, 0.059) (incr, 0.041) (kfw, 0.025) (eib, 0.023) (60, 0.013) (200, 0.012) (65, 0.011) (70, 0.011) (jul2009, 0.010)

Topic 66: (auto, 0.012) (sales, 0.011) (sees, 0.009) (stories, 0.008) (gm, 0.007) (car, 0.007) (europe, 0.006) (toyota, 0.005) (news, 0.005)

Topic 67: (percent, 0.032) (dollar, 0.013) (stocks, 0.010) (markets, 0.009) (index, 0.008) (shares, 0.008) (investors, 0.008) (asian, 0.008) (fell, 0.007) (yen, 0.007)

Topic 68: (05, 0.025) (04, 0.024) (pct, 0.019) (terms, 0.016) (06, 0.015) (yryr, 0.014) (2003, 0.013) (2004, 0.013) (03, 0.013) (oct, 0.013)

Topic 69: (nikkei, 0.019) (japan, 0.012) (market, 0.011) (stocks, 0.010) (yen, 0.008) (business, 0.007) (billion, 0.006) (friday, 0.006) (percent, 0.006) (daily, 0.005)

Topic 70: (2010, 0.036) (aug, 0.034) (world-bank, 0.030) (2009, 0.029) (na, 0.026) (jun, 0.024) (rand, 0.022) (mizuho, 0.020) (jul, 0.019) (feb, 0.019)

Topic 71: (reuters, 0.007) (company, 0.006) (business, 0.006) (million, 0.006) (billion, 0.005) (press, 0.004) (stories, 0.004) (companies, 0.004) (sony, 0.004) (executive, 0.003)

Topic 72: (yen, 0.047) (percent, 0.030) (billion, 0.015) (gmt, 0.015) (profit, 0.009) (year, 0.008) (million, 0.007) (shares, 0.007) (company, 0.007) (japans, 0.006)

Topic 73: (percent, 0.030) (yen, 0.023) (shares, 0.011) (nikkei, 0.011) (investors, 0.010) (earnings, 0.010) (market, 0.009) (rose, 0.006) (1, 0.006) (stocks, 0.006)

Topic 74: (yen, 0.045) (percent, 0.038) (gmt, 0.016) (profit, 0.009) (billion, 0.008) (shares, 0.007) (1, 0.006) (million, 0.005) (maker, 0.005) (forecast, 0.005)

Topic 75: (nihon, 0.012) (keizai, 0.012) (news, 0.011) (page, 0.011) (asahi, 0.011) (mainichi, 0.010) (yomiuri, 0.010) (japan, 0.010) (australian, 0.007) (japanese, 0.007)

Topic 76: (pct, 0.097) (100, 0.037) (incr, 0.035) (50, 0.035) (575, 0.026) (bng, 0.017) (55, 0.017) (200, 0.014) (frn, 0.011) (550, 0.011)

Topic 77: (percent, 0.038) (yen, 0.027) (nikkei, 0.013) (market, 0.011) (stocks, 0.010) (shares, 0.010) (1, 0.008) (investors, 0.007) (rose, 0.006) (fell, 0.006)

Topic 78: (yen, 0.048) (percent, 0.043) (gmt, 0.020) (shares, 0.011) (profit, 0.009) (billion, 0.009) (rose, 0.008) (1, 0.008) (nikkei, 0.006) (fell, 0.006)

Topic 79: (aaaaaa, 0.023) (rmbs, 0.019) (fixed, 0.018) (euro, 0.014) (100, 0.011) (frn, 0.011) (td, 0.010) (sec, 0.009) (aaa, 0.009) (aaaaaaaaa, 0.008)

Topic 80: (yen, 0.023) (percent, 0.018) (billion, 0.010) (gmt, 0.008) (profit, 0.008) (earnings, 0.008) (forecast, 0.006) (year, 0.005) (company, 0.005) (cut, 0.005)

Topic 81: (toyota, 0.022) (percent, 0.021) (year, 0.019) (000, 0.015) (sales, 0.014) (plant, 0.012) (car, 0.012) (million, 0.010) (production, 0.010) (units, 0.009)

Topic 82: (percent, 0.029) (points, 0.015) (index, 0.012) (new-york, 0.010) (gold, 0.009) (1, 0.007) (closed, 0.007) (prices, 0.006) (london, 0.006) (crude, 0.006)

Topic 83: (pct, 0.106) (100, 0.059) (incr, 0.035) (425, 0.017) (50, 0.015) (ontario, 0.014) (200, 0.013) (525, 0.011) (40, 0.010) (dec2007, 0.010)

Topic 84: (sales, 0.060) (percent, 0.032) (ford, 0.017) (year, 0.013) (toyota, 0.011) (auto, 0.011) (market, 0.010) (gm, 0.009) (vehicles, 0.009) (million, 0.008)

Topic 85: (profit, 0.028) (yen, 0.026) (billion, 0.025) (percent, 0.023) (year, 0.023) (toyota, 0.018) (sales, 0.015) (operating, 0.013) (forecast, 0.011) (earnings, 0.009)

Topic 86: (yen, 0.045) (percent, 0.035) (profit, 0.016) (gmt, 0.015) (billion, 0.014) (year, 0.010) (forecast, 0.008) (maker, 0.007) (sales, 0.006) (group, 0.006)

Topic 87: (pct, 0.104) (100, 0.031) (incr, 0.030) (200, 0.019) (50, 0.018) (rabobank, 0.016) (ge-capital, 0.015) (tranche, 0.014) (150, 0.011) (feb2012, 0.011)

Topic 88: (germany, 0.021) (1, 0.017) (ferrari, 0.017) (2, 0.014) (3, 0.014) (renault, 0.014) (toyota, 0.014) (britain, 0.014) (uk, 0.014) (4, 0.014)

Topic 89: (rmbs, 0.024) (fixed, 0.021) (frn, 0.017) (aaaaaa-, 0.013) (eurobond, 0.013) (aaaaaaaaa, 0.012) (anz, 0.010) (tap, 0.009) (200, 0.008) (2007-1, 0.008)

Topic 90: (yen, 0.038) (percent, 0.028) (billion, 0.013) (gmt, 0.012) (profit, 0.008) (group, 0.008) (maker, 0.007) (year, 0.007) (shares, 0.007) (million, 0.006)

Topic 91: (car, 0.010) (fiat, 0.010) (euro, 0.008) (renault, 0.008) (percent, 0.008) (europe, 0.005) (reuters, 0.005) (volkswagen, 0.005) (german, 0.005) (industry, 0.005)

Topic 92: (percent, 0.020) (yen, 0.020) (electronics, 0.015) (billion, 0.011) (nec, 0.009) (year, 0.009) (maker, 0.008) (sanyo, 0.007) (shares, 0.007) (corp, 0.007)

Topic 93: (words, 0.032) (moved, 0.021) (update, 0.016) (1, 0.010) (expect, 0.009) (2, 0.007) (london, 0.006) (600, 0.006) (gmt, 0.005) (3, 0.005)

Topic 94: (words, 0.036) (moved, 0.031) (pix, 0.012) (update, 0.011) (tv, 0.009) (1, 0.006) (600, 0.006) (president, 0.005) (2, 0.004) (700, 0.004)

Topic 95: (dec, 0.037) (nov, 0.037) (bonds, 0.021) (10, 0.018) (yen, 0.017) (mar, 0.013) (nomura, 0.013) (mizuho, 0.013) (date, 0.012) (na, 0.011)

Topic 96: (reuters, 0.019) (messaging, 0.017) (percent, 0.017) (stocks, 0.016) (click, 0.014) (shares, 0.014) (reutersnet, 0.011) (rm, 0.011) (double, 0.010) (stock, 0.010)

Topic 97: (steel, 0.065) (percent, 0.017) (nippon, 0.016) (prices, 0.015) (demand, 0.012) (year, 0.010) (price, 0.009) (¡5401t¿, 0.008) (nippon-steel, 0.007) (japanese, 0.007)

Topic 98: (bonds, 0.028) (yen, 0.021) (10, 0.017) (na, 0.014) (jul, 0.014) (date, 0.013) (oct, 0.012) (20, 0.010) (corp, 0.010) (mizuho, 0.010)

Topic 99: (pct, 0.035) (company, 0.024) (earnings, 0.020) (reported, 0.019) (toyota, 0.013) (expectations, 0.013) (year, 0.010) (corp, 0.010) (results, 0.010) (shares, 0.010)

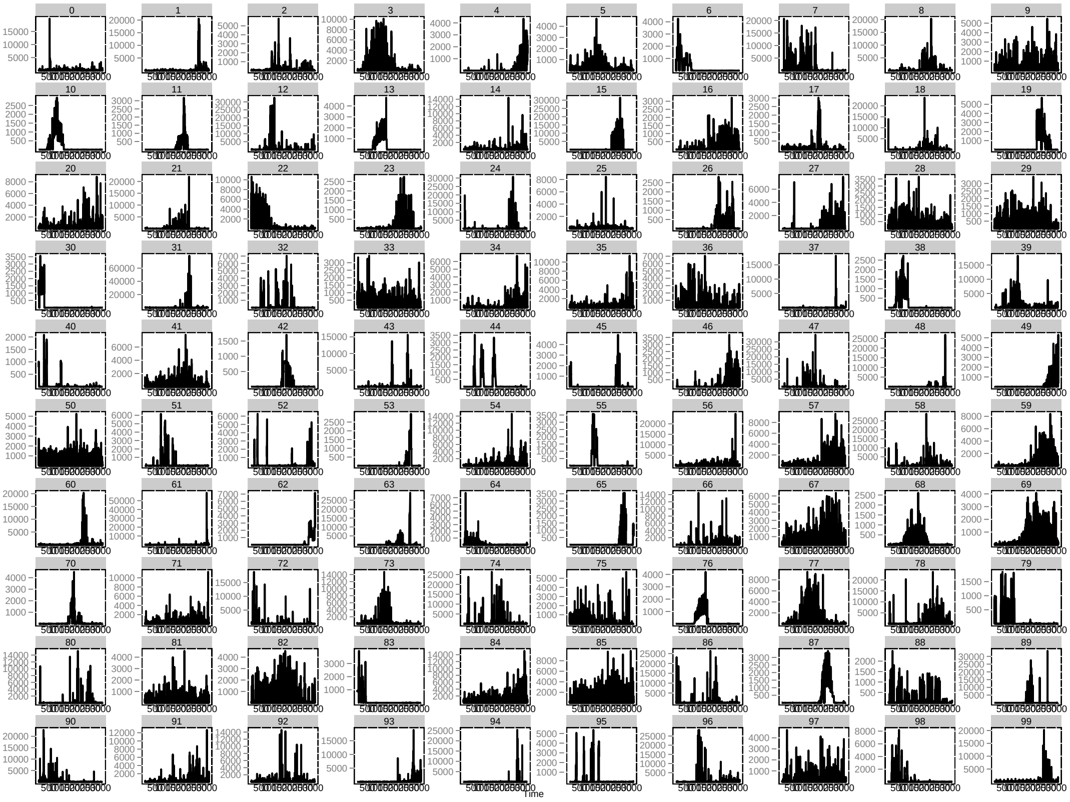

FIG. S1: Time evolution of news volume for all the learned topics for "Toyota". Horizontal axis denotes days (Jan 2003 to June 2011) and vertical axis shows their unnormalized volume.
